# Therapeutic education and physical activity are feasible and safe in hematologic cancer patients referred to chemotherapy: results of a randomized controlled trial

**DOI:** 10.1007/s00520-022-07530-4

**Published:** 2022-12-19

**Authors:** Monia Allisen Accogli, Monica Denti, Stefania Costi, Stefania Fugazzaro

**Affiliations:** 1 Physical Medicine and Rehabilitation Unit, Azienda Unità Sanitaria Locale-IRCCS Di Reggio Emilia, Viale Risorgimento N°80, 42123 Reggio Emilia, Italy; 2 Department of Surgery, Medicine, Dentistry and Morphological Sciences, University of Modena and Reggio Emilia, Via del Pozzo N°74, 41100 Modena, Italy

**Keywords:** Hematologic neoplasms, Exercise, Physical therapy modalities, Patient education, Rehabilitation, Non-pharmacologic treatments

## Abstract

**Purpose:**

Although over 60% of patients with hematologic cancer report distressing fatigue, they often do not receive recommendations on fatigue management strategies. The aim of this pilot study was to estimate the feasibility of therapeutic education and physical activity (TEPA) by measuring the patients’ adherence to this multidimensional intervention. The secondary aim was to estimate the impact of TEPA on clinical outcomes.

**Methods:**

Patients with hematologic cancer participated in this single-center, open-label, randomized controlled trial. The control group (CG) received two educational group sessions on fatigue and physical activity. The experimental group (EG) received the two educational sessions plus six weekly individual sessions aimed at implementing a personalized physical exercise program. Follow-ups were at 1, 3, and 7 months.

**Results:**

Forty-six patients referred to chemotherapy were included, corresponding to 54% of recruitment rate. Adherence reached 90% in the EG and 68% in the CG. Most patients (65% in EG and 64% in CG) attended a minimum of 80% of the planned sessions. Overall retention rate was 87% (85% in EG and 91% in CG). No adverse events were registered. No between-group differences were detected in fatigue (FACIT-F), psychological distress (NCCN Distress Thermometer), QoL (EORTC QLQ-C30), or functional exercise capacity (TUG test and 6MWT). Adherence to an active lifestyle, measured by a semi-structured interview, increased from 56.5 to 84% in the EG at 7 months (*p* = 0.02), whereas it decreased slightly in the CG (from 47.8 to 42.9%).

**Conclusion:**

Multidimensional rehabilitation interventions are feasible and safe in this population, and larger trials should focus on the efficacy of such approaches on clinically relevant outcomes.

**Trial registration:**

ClinicalTrials.gov Identifier: NCT03403075.

**Supplementary information:**

The online version contains supplementary material available at 10.1007/s00520-022-07530-4.

## Introduction

Hematologic malignancies account for nearly 8% of new cancer diagnoses in Italy [1]. Their clinical course varies, and treatment can consist of radiotherapy, chemotherapy, immunotherapy, and/or autologous or allogeneic stem cell transplant. These therapeutic approaches can cause long-term side effects, such as physical deconditioning, cancer-related fatigue (CRF), and worsening of quality of life (QoL).

CRF has been defined as “a distressing, persistent, subjective sense of tiredness or exhaustion related to cancer or cancer treatment which is not proportional to recent activity and interferes with usual functioning” [2]. CRF is reported by 60–90% of patients with hematologic malignancies [3]. It frequently persists long after the end of treatment, impacting on physical performance and QoL. CFR has a multifactorial etiology and is intensified by the comorbidities that characterize hematologic malignancies, such as sarcopenia, anemia, and neutropenia [4].

From a clinical point of view, CRF provokes a vicious circle of physical deconditioning and limitations in activities, which in turn increase fatigue and cause further limitations and mood disturbances [5]. Moreover, physical deconditioning is highly prevalent in individuals with hematologic malignancies, as it also derives from side effects of treatments, such as cardiotoxicity, neurotoxicity, or cachexia, and from the widespread belief that the patient should rest in order to cope with CRF [6]. Physical deconditioning has also been associated with a worse response to cancer treatment [7].

Physical activity and therapeutic education have been recommended in this population at any cancer stage to reduce fatigue [3, 8, 9]. In fact, physical activity is safe and improves physical functioning, CRF, QoL, and mood disturbances in patients with hematologic malignancies [10]. In particular, aerobic exercise improves cardiorespiratory fitness, physical well-being, and muscle strength in this population [3]. Moreover, multidimensional programs that combine physical activity and therapeutic education may be particularly useful in managing the multifaceted symptoms of CRF, both during and after treatment [11, 12]. A better management of CRF increases the tolerance to physical activity and may interrupt the vicious circle that sustains progressive physical deconditioning [3, 5, 7, 9]. However, patients often receive vague information and no personalized support regarding recommended exercise during the course of cancer and its treatment [11, 13].

Brief, focused, multidimensional rehabilitation interventions including therapeutic education and physical activity may be particularly useful to manage CRF [11, 12, 14], but the most appropriate contents and delivery modalities still need clarification [12]. To date, such multidimensional rehabilitation interventions have been studied for their effectiveness in populations at any stage of cancer, but only few studies have examined individuals with hematologic malignancies [11].

We therefore implemented a multidimensional rehabilitation intervention that includes therapeutic education and physical activity (TEPA) to address the needs of individuals with hematologic malignancies. A randomized controlled trial was conducted to verify the feasibility and impact of the TEPA intervention in this population.

## Materials and methods

### Study design

This was a pilot feasibility unblinded single-center randomized controlled trial with two parallel groups. The protocol of this study was approved by the Provincial Ethics Committee of Reggio Emilia (prot. n. 2017/0071458, July 19, 2017) and prospectively registered on ClinicalTrials.gov [15].

The trial took place at the Santa Maria Nuova Hospital (SMN) of the Azienda Unità Sanitaria Locale-IRCCS di Reggio Emilia (Italy) and is reported according to the CONSORT checklist [16].

### Participants

Adult patients (≥ 18 years) referred to the Hematology Unit were considered eligible if affected by a first or an early relapse of hematologic malignancy and candidate to chemotherapy and/or radiotherapy, but not yet undergoing treatment. We excluded individuals with poor prognosis (< 12 months) and clinical conditions that may hinder participation in the rehabilitation program (e.g., dementia, psychiatric pathology, blindness). Prognosis was estimated by the referring hematologists according to the severity of the hematologic malignance, the expected response to cancer treatments, and the estimated survival at 12 months.

Written consent was collected from all participants.

### Interventions

The study implemented two parallel active multidimensional rehabilitation interventions which were administered in addition to usual care, i.e., the treatment regimen prescribed by the referring hematologist. Both the interventions have already been described in detail in the study protocol by Denti and colleagues [17].

The control group was exposed to the therapeutic education (TE) intervention. It consisted in two educational small-group sessions which lasted 1 h each, were held at the hospital, were led by two trained physical therapists, and were open to caregivers. The groups consisted in a minimum of two up to six participants. These two educational sessions shared a common structure but differed in their main focus, which was CRF management for one session and physical activity for the other. The sessions were not delivered in any specific order, so that eligible patients and their caregiver could start with either of them. The contents were addressed through lectures, group activities, and brainstorming, with the support of an interactive whiteboard and a projector. The key concepts of the group sessions were summarized in a brief handout that was provided to participants.

The experimental group was exposed to the TEPA intervention, which consisted in the TE intervention provided to the control group plus six face-to-face individual sessions that lasted about 20 min each, were held at the hospital, led by trained physical therapists, and were scheduled once a week or every 2 weeks. During the individual sessions participants could discuss more in depth with the trained physical therapist the topics addressed in the group sessions, and they developed a personalized weekly physical activity program called the Activity Plan. The Activity Plan, tailored to the participant’s clinical condition and preferences, had to be performed independently in between the individual sessions through the counselling provided by the physical therapist, who assisted participants in planning their weekly physical activities, checked the adherence to these activities, and supported patients in achieving their goals. The key concepts of the individual sessions were summarized in a short written handout that was provided to participants, together with a list of the suggested exercises and the activity diary, where participants recorded the physical activity that they performed independently.

### Outcome measures and assessments

The primary aim of this study was to investigate the feasibility of the TEPA intervention in individuals with hematologic cancer.

Sociodemographic and clinical data were collected at baseline (T0), which took place after diagnosis and before the beginning of lifesaving treatments; follow-up assessments took place at 1 month (T1), 3 months (T2), and up to 7 months (T3) from baseline. The TEPA intervention was proposed between T1 and T2.

Feasibility was assessed through intervention completion by measuring the degree of adherence between the planned intervention and the actual intervention, i.e., comparing the number of sessions completed/recorded with those planned for each participant. We also estimated the feasibility of the intervention by collecting data on its safety and by recording the recruitment and retention rates.

Safety was assessed by tracing the number and type of adverse events (AEs) possibly related to physical activity, such as fractures or injuries, musculoskeletal pain, muscle cramp, and falls. The physiotherapists asked patients to refer any adverse event after every weekly individual session, including whether the AE required medical attention.

The recruitment rate was calculated as the ratio between the randomized participants and the eligible individuals. The retention rate was calculated as the ratio between the participants that completed the study and those randomized.

The secondary aims were to estimate the effect size of the TEPA intervention on clinically relevant outcomes and to estimate its educational impact, that is, the degree to which the TEPA can affect behavior. The effect size of the TEPA intervention was estimated by measuring the changes in:

- (a) cancer-related fatigue, measured by the FACIT Fatigue Scale [18]. This is a self-reported 13-item tool that measures an individual’s level of fatigue on a 0 to 4 rating scale during usual daily activities over the previous week. Higher scores represent higher levels of fatigue.
- (b) psychological distress, measured by the NCCN Distress Thermometer and Problem List for Patients [19]. This is a self-reported tool which consists in a single item using a 0 (no distress) to 10 (extreme distress) rating scale to assess distress over the previous week, integrated with a 39-item list of potential sources of distress.
- (c) QoL, measured by the EORTC Quality of Life Questionnaire-C30 [20]. It is a self-reported 30-item tool including subscales for functional status, symptoms, and global health. The subscales range in score from 0 to 100, with higher scores representing higher response level to any scale.
- (d) physical performance (mobility, balance, walking ability), measured by the Timed Up & Go (TUG) test [21]. It is a field test that measures the time needed to stand up from a seated position, walk forward 3 m as quickly as possible, turn around, walk back to the chair, and sit down.
- (e) functional exercise capacity, measured by the Six-Minute Walk Test (6MWT) [22]. This field test measures the distance in meters a person can walk in 6 min. It was administered according to the American Thoracic Society guidelines for administration and clinical interpretation [22].

The three questionnaires and the TUG were administered at all the assessment times, while, for feasibility reasons, the 6MWT was administered only at baseline and at T3.

The educational impact of the TEPA intervention was estimated by testing the first and the third levels of the Kirkpatrick Model [23]: the reaction was estimated through semi-structured interviews that collected data regarding the participants’ satisfaction and perception of the usefulness of the intervention provided (ESM Appendix A); behavior was estimated by collecting data on the participants’ long-term adherence to an active lifestyle (ESM Appendix B).

Assessments were performed by a trained physical therapist, following the schedule presented in ESM Appendix C.

Due to the nature of the intervention, only T0 assessment could be carried out blind to participants’ allocation to the experimental or control group. However, to minimize any possible performance bias, the two arms of the study were both subjected to active treatments in addition to usual care.

### Sample size and randomization procedures

As there were no data from previous trials to rely on, in the absence of an a priori hypothesis for dimensioning, the sample size was established according to the number of new hematologic malignancies diagnosed per year at the Hematology Unit of the SMN Hospital. We assumed that a sample of 40 participants, 20 per group, would be acceptable for a pilot study [17].

After the first blinded assessment (T0), participants were randomized to the control or experimental group, in a 1:1 allocation ratio. Random allocation sequence was generated and managed by the Clinical Trial and Statistics Unit of the Azienda Unità Sanitaria Locale-IRCCS di Reggio Emilia (not involved in patients’ treatment or evaluation); to ensure allocation concealment, researchers were informed of group allocation for each patient by telephone call.

### Statistical analysis

Data were collected electronically in aggregated and anonymous modalities. All analyses were intention-to-treat and conducted using all data available (complete case analysis). A secondary per-protocol analysis was planned if adherence did not reach the cutoff of 80% in the experimental group; however, this was not performed, given the obtained results.

Clinical and demographic data are expressed as frequencies and percentages for categorical variables and as mean and standard deviation for quantitative variables.

Regarding the primary aim, the proportion estimates of sessions completed to those planned are accompanied by Wilson confidence intervals.

For the effect size of TEPA, we compared groups (both for point estimates at given times and for T3-T0 variation) using two independent sample t-tests and estimating mean difference with confidence interval, assuming a normal distribution of the estimator.

Finally, adherence to an active lifestyle is described by percentage estimates and their associated confidence intervals according to Wilson and compared by Chi-square tests. The level of satisfaction and perception of the usefulness of the provided interventions were analyzed descriptively (percentages).

All confidence intervals were two-tailed and calculated considering a 0.95 confidence level. Statistical analyses were performed using R 3.3.2 [24].

## Results

**Fig. 1:**
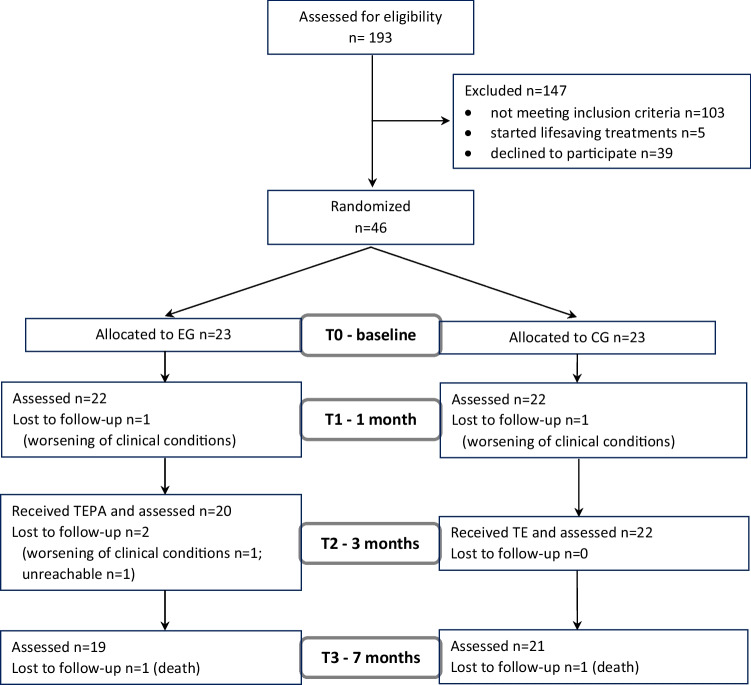
Flowchart of study participants

From March 2018 to March 2019, 193 potentially eligible individuals were screened for eligibility. Figure 1 shows the flow of study participants of this study.

We excluded 103 individuals who did not meet the inclusion criteria, and five individuals could not be enrolled since at the time of the screening they had already started lifesaving treatments. Eighty-five eligible individuals were completely informed about the study, and 39 refused to participate. Thus, 46 individuals consented to participate, all referred to chemotherapy: 23 were randomly assigned to the experimental group and 23 to the control group.

The sociodemographic and clinical characteristics of the participants are reported in Table 1. Participants enrolled in the two groups were similar at baseline for the main prognostic variables that were measured. On average, participants were 59.9 years old, with almost 12 years of education. At study enrolment, most participants (*n* = 39, 84.8%) were referred to the Hematology Unit as outpatients. The most represented diagnosis in the sample was lymphoma (*n* = 30, 65.2%).

**Table 1:** Sociodemographic and clinical characteristics

Variables	CG (*n* = 23)	EG (*n* = 23)	Total (*n* = 46)	*p*	
Age, median [IQR]	60.39 [49.96, 67.46]	66.66 [51.30, 72.14]	61.76 [49.83, 70.61]	0.350	§
Sex, *n* (%)					
Male	9 (39.1)	15 (65.2)	24 (52.2)	0.140	¥
Female	14 (60.9)	8 (34.8)	22 (47.8)		
Years of education, median [IQR]	8.00 [8.00, 12.50]	13.00 [9.00, 13.50]	11.00 [8.00, 13.00]	0.084	§
Occupation, *n* (%)					
Employed	10 (43.5)	8 (34.8)	18 (39.1)	0.408	¤
Unemployed	5 (21.7)	2 (8.7)	7 (15.2)		
Retired	7 (30.4)	12 (52.2)	19 (41.3)		
Other	1 (4.3)	1 (4.3)	2 (4.3)		
Household, *n* (%)					
Live alone	1 (4.3)	0 (0.0)	1 (2.2)	1.000	¤
Live with others	22 (95.7)	23 (100.0)	45 (97.8)		
Hospitalization regime, *n* (%)					
Outpatient	19 (82.6)	20 (87.0)	39 (84.8)	1.000	¤
Inpatient	4 (17.4)	3 (13.0)	7 (15.2)		
Diagnosis, *n* (%)					
Lymphoma	17 (73.9)	13 (56.5)	30 (65.2)	0.395	¤
Leukemia	2 (8.7)	5 (21.7)	7 (15.2)		
Multiple myeloma	4 (17.4)	5 (21.7)	9 (19.6)		
Hemoglobin, median [IQR]	12.00 [10.07, 13.05]	11.50 [10.50, 13.35]	11.80 [10.30, 13.10]	0.742	§

^§^Mann–Whitney *U* test, ¥Chi2 test, ¤Fisher’s exact test

### Feasibility data

Eighty-five patients out of 193 matched the inclusion criteria. Forty-six consented to participate, corresponding to 54% of the recruitment rate. Six participants were lost to follow-up, corresponding to 87% of the retention rate: four in the experimental group (85% of retention rate) and two in the control group (91% of retention rate). The reasons for dropping out of the study were the worsening of clinical condition (n = 3) or death (n = 2), with one individual unreachable from T2. None of these reasons could be associated with the interventions under study.

No adverse events were registered during the study.

The degree of adherence to the interventions was assessed between T1 and T2. Data were available for 20 participants in the experimental group and 22 in the control group.

A proportion of 64% of participants (*n* = 14) in the control group and 65% of participants (*n* = 13) in the experimental group attended a minimum of 80% of the planned sessions (two sessions for TE intervention and eight sessions for TEPA intervention).

Overall, adherence to TE was 68% of the 44 educational small-group sessions, while adherence to the TEPA intervention reached 90% of the 160 planned sessions (95% CI: 84%—94%): 100% adherence to the 120 individual sessions and 60% adherence to the educational small-group sessions.

Reasons for not participating in the sessions were hospitalization (14% in CG and 63% in EG), refusal / unwillingness to participate (50% in CG and 6% in EG), and organizational or transportation problems (36% in CG and 31% in EG).

Overall, the 17 participants that missed at least one group session had the following diagnosis: lymphoma (*n* = 12), leukemia (*n* = 4), myeloma (*n* = 1).

### Exploratory effect sizes of TEPA

**Fig. 2:**
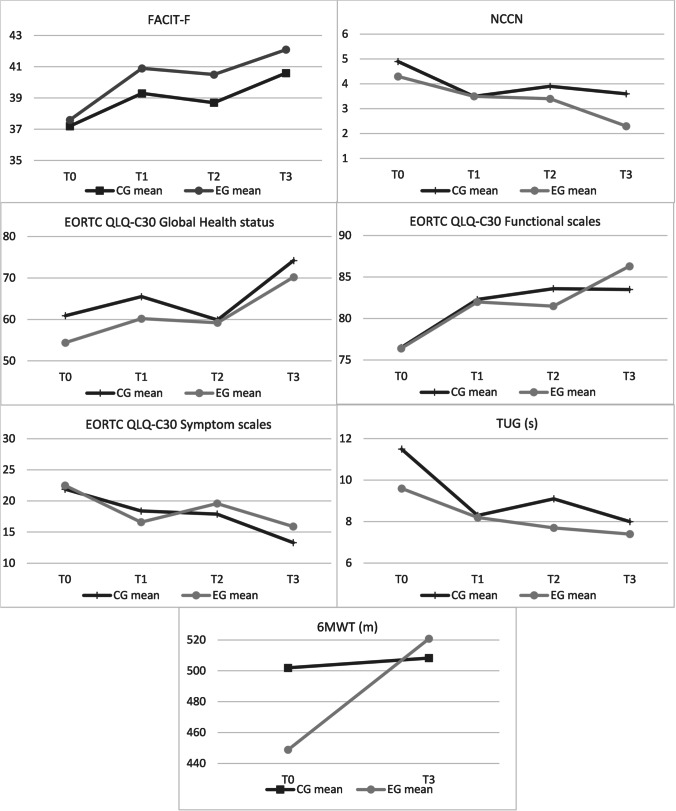
Improvements in clinical outcomes shown by both the arms in study

The changes at follow-up in the clinical secondary outcomes of this study are shown in Tables 2 and 3 and Fig. 2. Details of comparisons are presented in ESM Appendix D. The results show no statistically significant differences between the two groups. Cancer-related fatigue did not differ, with a clinically relevant improvement detected in the EG, since the mean change from T0 to follow-up exceeded the minimal clinically important difference of three points for the FACIT-F [25].

**Table 2:** Changes in clinical outcomes over time

Outcomes	Measures	Group	T0 Mean (SD)	T1 Mean (SD)	T2 Mean (SD)	T3 Mean (SD)
CRF	FACIT-F	CG	37.2 (10.5)	39.3 (9.2)	38.7 (9.6)	40.6 (7.8)
		EG	37.6 (10.6)	41 (7.0)	40.6 (7.1)	42.1 (6.2)
Emotional distress	NCCN Distress Thermometer	CG	4.9 (3.1)	3.5 (2.7)	3.9 (2.8)	3.6 (2.6)
		EG	4.3 (2.8)	3.5 (2.2)	3.4 (2.1)	2.3 (2.2)
QoL – Global Health Status	EORTC QLQ-C30	CG	60.9 (24.4)	65.5 (24.5)	59.8 (22.2)	74.2 (16.7)
		EG	54.3 (25.4)	60.2 (23.3)	59.2 (25.1)	70.2 (17.2)
QoL – Functional Scales	EORTC QLQ-C30	CG	76.5 (18.5)	82.3 (13.7)	83.7 (11.5)	83.5 (12.9)
		EG	76.4 (18.9)	82.0 (14.5)	81.5 (14.1)	86.3 (12.6)
QoL – Symptom Scales	EORTC QLQ-C30	CG	21.9 (15.3)	18.4 (14.1)	18.0 (13.4)	13.3 (7.3)
		EG	22.5 (17.1)	16.6 (14.1)	19.6 (13.9)	15.9 (12.7)
Physical performance	TUG test (s)	CG	11.5 (8.9)	8.3 (2.4)	9.1 (3.3)	8.0 (2.4)
		EG	9.6 (3.6)	8.2 (2.5)	7.7 (1.7)	7.4 (2.1)
Physical performance	6MWT (m)	CG	501.9 (116.6)	-	-	508.3 (96.0)
		EG	448.9 (111.5)	-	-	520.8 (90.6)

**Table 3:** Between-group comparisons

	T0–T3				
Measures	CG	EG	Diff	CI	*p*
FACIT-F	1.9 (11.6)	3.6 (10.2)	1.7	− 5.4 to 8.7	0.633
NCCN Distress Thermometer	− 0.9 (2.9)	− 1.9 (2.2)	− 1	− 2.6 to 0.7	0.232
EORTC Global Health Status	10.7 (25.7)	14 (26.4)	3.3	− 13.4 to 20	0.689
EORTC Functional Scales	3.9 (16.9)	8.8 (15.9)	4.9	− 5.7 to 15.4	0.356
EORTC Symptom Scales	− 6.2 (13.1)	− 7 (15.5)	− 0.8	− 10 to 8.3	0.854
TUG test	− 1.1 (2.1)	− 1.8 (2.7)	− 0.7	− 2.4 to 1	0.408
6MWT	12.4 (72.8)	36.3 (44.9)	23.9	− 26.1 to 73.9	0.334

Psychological distress decreased during the study, mostly in EG, albeit without statistical significance. The same trend was seen in the number of problems reported by the participants, which progressively decreased over the 7 months of follow-up.

All the QoL subdomains improved over the course of the study in both groups, but no statistically significant difference was detected in the global EORTC score between the groups at any time.

As regards physical performance, there was a trend towards improvement in mobility, balance, walking ability, and fall risk. Several participants could not perform the TUG test at some of the assessment times due to concomitant administration of intravenous therapy (*n* = 8), suboptimal clinical condition (e.g., pain, fatigue) (*n* = 5), or protective isolation after stem cell transplant (*n* = 2). Although not significant, it is worth noting that walking ability improved by 71.9 m in the experimental group and by 6.4 m in the control group. Again, it was not always possible to administer the 6MWT due to the need for protective isolation of patients (*n* = 12).

Figure 2 illustrates the trend of clinical variables measured in the two groups for the duration of the study.

### Educational impact of TEPA

Patient satisfaction and perception of usefulness of the intervention provided were estimated on 35 participants, 16 in the control group and 19 in the experimental group. Overall, most participants reported satisfaction with the amount of information received (74%); the information received was judged sufficiently (38% CG, 21% EG) or very useful (63% CG, 79% EG) by all the respondents. Moreover, 66% of participants considered themselves as very knowledgeable about disease self-management strategies and behaviors to be implemented at home in order to facilitate recovery (e.g., energy conservation techniques strategies, management of social relationships, physical activity). Nevertheless, 11% of participants would have liked to have received more information, especially regarding nutrition, movement, sexual activity, and their disease.

The participants’ long-term behavior, i.e., their adherence to an active lifestyle at follow-up, is described in Table 4.

**Table 4:** Long-term adherence to active lifestyle

Physical activity carried out regularly						
	Baseline (T0)			Follow-up (7 months)		
**Group**	***n***	**Yes (*n*,%)**	**95% confidence interval**	***n***	**Yes (*n*,%)**	**95% confidence interval**
CG	23	11 (47.8%)	27.4–68.9	21	9 (42.9%)	22.6–65.6
EG	23	13 (56.5%)	34.9–76.1	19	16 (84.2%)	59.5–95.8

If, at the beginning of the study, about half of the participants in both groups (47.8% control, 56.5% experimental, Chi2 test *p*-value = 0.8) reported having an active lifestyle, at the final follow-up approximately twice as many participants in the experimental group (84.2%) as in the control group (42.9%) continued to exercise regularly (*p*-value = 0.02).

## Discussion

This study compared two active multidimensional rehabilitation interventions offered to patients with hematologic cancer and collected data regarding the feasibility of these types of interventions in patients with hematologic cancer, a population that is underrepresented in studies on cancer rehabilitation [3]. Overall, this study showed good adherence to the experimental intervention, since participants allocated to this group attended all the individual sessions of the TEPA intervention and 60% of the small-group sessions. The recruitment rate exceeded 50% and the retention rate was high (85% in the experimental group and 91% in the control group); moreover, no adverse events associated with the experimental intervention were reported during the study period. Thus, this study showed that the TEPA intervention was feasible and safe.

Participant adherence to the TEPA intervention reached 90%, clearly exceeding the cutoff of 80% set a priori. Despite the lower commitment required, the adherence to the TE intervention reached 68%.

Adherence to treatment is a pivotal issue in cancer rehabilitation and can be influenced by several factors. This study confirmed some predictors of adherence to exercise that were already noted in previous research [26]; being male and having family support may promote participation in exercise programs. In the sample we examined, 65.2% of the participants in the experimental group were male, compared to 39% in the control group, which may account for the better adherence to exercise in former group. Another predictor of adherence is the distance from a patient’s home to the facility where the interventions take place; in the sample we examined, more than one third of participants did not attend some sessions because of transportation issues. Adherence to exercise is also influenced by clinical characteristics, such as the presence of CRF at baseline, the advanced stage of the disease, or a cancer treatment that impacts performance status [26]. In this study, patients with lymphoma, leukemia, or multiple myeloma were treated according to the recommended approaches and timing. The individual sessions that characterized the TEPA intervention were tailored to each individual’s needs and clinical pathway. Thus, the six individual sessions resulted completely feasible, even when held during high-dose chemotherapy. Instead, adherence to the standardized educational small-group session was 60% and 68% in the experimental and the control groups, respectively.

The secondary aim of this study was to explore the impact of the TEPA intervention on fatigue, psychological distress, quality of life, and physical performance. In addition, we estimated the educational impact of the TEPA intervention on the patients’ satisfaction and perception of the usefulness of the intervention provided [23] and on their long-term adherence to an active lifestyle after cancer diagnosis [23].

This pilot study was not powered to detect any statistically significant differences between the groups in any of the clinically relevant outcomes measured, and in fact, no significant differences were detected between the study groups in terms of fatigue, QoL, or psychological distress; however, all these outcomes showed similar trends towards improvement in both groups, which were both exposed to active multidimensional rehabilitation interventions. Of note, the improvement achieved by participants in the EG in CRF was clinically relevant, as the mean change from T0 to follow-up exceeded the cutoff of minimal clinically important difference set for the FACIT-F [25]. For all the other outcome measures used, the cutoffs of minimal clinically important difference have not been established in hematologic patients.

Some considerations must be made to better interpret these results. A recent Cochrane review by Knips et al. concluded that physical exercise added to standard care might improve fatigue and depression in hemathologic patients, but the evidence was inconclusive regarding other relevant outcomes, such as quality of life, physical functioning, anxiety, and mortality [3]. The same review also called for further research with high internal validity to determine the true effect of physical activity on relevant outcomes and to define the most appropriate exercise dose and modality according to cancer stage and treatments.

Since the study design did not include a placebo group, we cannot exclude that the favorable changes observed were due to the exposure to the active interventions of both the groups in study. Regarding the changes in physical performances, although they did not differ between groups, it should be highlighted that the improvement in the distance walked in 6 min at T3 was much greater in the experimental group (72 m) than in the control group (6 m); this difference far exceeds the minimum clinically important distance defined for the 6MWT, which has been estimated to be between 22 to 42 m in individuals with lung cancer [27] or 29 m in a population that included individuals with hematologic malignancies treated with bone marrow transplantation [9]. Future larger studies should investigate whether these types of interventions can ameliorate physical performance.

Also of value is the consideration of the applicability of the physical performance measurements included in this study, because not all proved feasible in the very first months of lifesaving treatments for hematologic cancer. This was particularly true for the 6MWT, which is a reliable instrument to describe physical performances in cancer patients and which correlates to EORTC QLQ-C30 physical function subscale. However, this measurement is not easily administered when patients are isolated after having undergone stem cell transplant or after high-dose chemotherapy. In our sample, only 30 of the 46 participants enrolled could perform the test at baseline, adversely affecting the possibility of detecting any possible statistically significant change. Conversely, the TUG test [21] turned out to be less time-consuming and simpler to administer in the hospital setting, even when participants were isolated. Thus, even if the TUG test is not the gold standard measurement for exercise capacity, it represents a valuable option.

Finally, long-term adherence to an active lifestyle was significantly higher in the experimental group, with 84% of participants exercising regularly 7 months after cancer diagnosis compared to 43% in the control group. The supervised one-to-one sessions with the physiotherapists facilitated hematologic patients’ adherence to regular exercise at follow-up. This means that the individuals exposed to the TEPA intervention continued to apply what they had learned for months after stopping the intervention. If confirmed in future studies, this result is probably the one with the greatest potential in terms of prevention.

After being diagnosed with a hematologic malignancy, patients are in a ‘teachable moment’ [28]. Clinicians should thus take advantage of this moment and educate patients about the advantages of adopting healthier behaviors. Since advances in cancer treatments increase the prevalence of cancer survivors, this education may be crucial in reducing the risk of secondary malignancies or the risk of developing chronic comorbidities [28]. If we consider exercise as a drug, researchers should investigate the appropriate dosage in terms of frequency, modalities, intensity, and duration according to cancer stage and treatment, and rehabilitation professionals should give patients precise recommendations in terms of the exercise needed to achieve specific health outcomes.

This trial has some limitations. First, since the study design did not include a placebo group that was not involved in rehabilitation, it is possible that the improvements in clinically relevant outcomes detected in both active intervention arms were due to the natural history of the disease. Moreover, because this trial was conducted in a single center, the generalizability of its findings is limited. The lack of blinding is another limitation, but the outcomes considered were unlikely to have been influenced, as most assessments were patient-driven, while performance tests were conducted according to international recommendations. Finally, this was a pilot exploratory study whose sample size was based mainly on local feasibility. Thus, it does not provide definitive information on the effect size of the experimental intervention.

However, among its strengths, this study implemented two active rehabilitation interventions that included strategies to support adherence to exercise, such as personalization of the intervention according to each participant’s needs and clinical pathway, goal setting, written education material, and social support within group sessions, as suggested by a recent review [29].

## Conclusions

To our knowledge, this is the first study to assess the feasibility of a rehabilitation intervention that includes exercise and education implemented in the very early phase (or early relapse) of hematologic cancer.

Even if, as already mentioned, this pilot study cannot give indications on the impact of the active interventions tested on clinically relevant outcomes, it confirmed the feasibility and safety of the rehabilitation interventions described and suggests that, in the first months after a diagnosis of hematologic cancer, individual sessions can be implemented more easily compared to small-group sessions. Moreover, this study highlighted a significant difference in long-term adherence to an active lifestyle in participants exposed to individual exercise sessions. Thus, the results of this study can be useful to other researchers when designing larger trials that focus on the efficacy of multidimensional rehabilitation approaches to address clinically relevant outcomes of hematologic cancer patients.

## Supplementary Information

Below is the link to the electronic supplementary material.

Supplementary file1 The full protocol of this trial has beenpreviously published in open access: Denti M, Accogli MA, Costi S, Fugazzaro S.Therapeutic Education and Physical Activity to Support Self-management ofCancer-related Fatigue in Hematologic Cancer Patients: Protocol of aFeasibility Randomized Controlled Trial. Integr Cancer Ther.2020;19:1534735420969830. doi:10.1177/1534735420969830. (DOCX 24 KB)

Supplementary file2 (DOC 220 KB)

## Data Availability

Dataset generated and analyzed in the current study was managed by the Information and Technologies Service (STIT) of the Azienda USL-IRCCS of Reggio Emilia in order to protect patient privacy. The data that support the findings of this study are available from the corresponding author upon reasonable request.
