# Supplementary material for: Therapeutic education and physical activity are feasible and safe in hematologic cancer patients referred to chemotherapy: results of a randomized controlled trial
Source: Support Care Cancer. 2022 Dec 19;31(1):61. doi: 10.1007/s00520-022-07530-4 (PMC9763142; doi:10.1007/s00520-022-07530-4)
Supplement: Supplementary file 1 — Supplementary file1 The full protocol of this trial has beenpreviously published in open access: Denti M, Accogli MA, Costi S, Fugazzaro S.Therapeutic Education and Physical Activity to Support Self-management ofCancer-related Fatigue in Hematologic Cancer Patients: Protocol of aFeasibility Randomized Controlled Trial. Integr Cancer Ther.2020;19:1534735420969830. doi:10.1177/1534735420969830. (DOCX 24 KB) [file 520_2022_7530_MOESM1_ESM.docx]

**Appendix A.**

Semi-structured interviews to collect data regarding the participants’ satisfaction and perception of the usefulness of the intervention provided

|  | STRONGLY AGREE | SOMEWHAT DISAGREE | SOMEWHAT AGREE | STRONGLY AGREE |
| --- | --- | --- | --- | --- |
| The amount of information received was satisfactory | 1 | 2 | 3 | 4 |
| Overall, the information received was useful | 1 | 2 | 3 | 4 |
| During the course of meetings have been well informed on: |  | | | |
| Manage the disease at home | 1 | 2 | 3 | 4 |
| What actions or behaviors help to recover (ex. rest, social relationships, physical activity ...) | 1 | 2 | 3 | 4 |

| Would you have preferred to receive further information? | - Yes | - No |
| --- | --- | --- |
| If so, specify on which topics  ______________________________________________________________________________ | | |
| Would you have preferred to receive less information? | - Yes | - No |
| If so, specify on which topics  _____________________________________________________________________________ | | |

**Appendix B.**

Semi-structured interviews to collect data regarding the participants’ long-term adherence to an active lifestyle

| Do you performe Physical activity regularly? | € Yes € No |
| --- | --- |
| If so, what kind of activity? ________________________________________________________________ | |
| How often?   - Everyday - 2-3 times a week - Once a week - Other ____________________ | How long? (duration)   - < 30 min - 30 min - 1 hour - > 1 hour - Other ___________________ |
| - Alone - In a group | Notes  __________________________________________ |

**Appendix C.** Outcome measures and assessment schedule.

| **Outcomes** | **Measures** | **T0** | **T1** | **T2** | **T3** |
| --- | --- | --- | --- | --- | --- |
| Sociodemographic and clinical characteristics^a^ | Anamnesis, data from medical record | X |  |  |  |
| Blood chemistry tests^b^ | Laboratory test | X | X | X | X |
| CRF | FACIT-F | X | X | X | X |
| Psychological distress problem list | NCCN Distress Thermometer | X | X | X | X |
| QoL | EORTC QLQ-C30 | X | X | X | X |
| Physical performance | TUG | X | X | X | X |
|  | 6MWT | X |  |  | X |
| Type and amount of physical activity performed regularly | Semi-structured interview | X |  |  | X |
| Participant satisfaction and perception of usefulness of the intervention provided | Semi-structured interview |  |  |  | X |

^a^Sex, age, years of education, occupation, living condition. ^b^The most recent blood chemistry tests were collected at each assessment time.

**Appendix D.** Outcome analysis between groups

|  | **T0** | | | | | **T1** | | | | | **T2** | | | | | **T3** | | | | |
| --- | --- | --- | --- | --- | --- | --- | --- | --- | --- | --- | --- | --- | --- | --- | --- | --- | --- | --- | --- | --- |
| **Measure** | **CG** | **EG** | **Diff** | **CI** | **p** | **CG** | **EG** | **Diff** | **CI** | **p** | **CG** | **EG** | **Diff** | **CI** | **p** | **CG** | **EG** | **Diff** | **CI** | **p** |
| FACIT-F | 37.2 (10.5) | 37.6 (10.6) | 0.3 | -5.9 to 6.6 | 0.912 | 39.3 (9.2) | 41 (7) | 1.7 | -3.4 to 6.7 | 0.506 | 38.7 (9.6) | 40.6 (7.1) | 1.9 | -3.5 to 7.2 | 0.482 | 40.6 (7.8) | 42.1 (6.2) | 1.5 | -3 to 6 | 0.509 |
| NCCN Distress Thermometer | 4.9 (3.1) | 4.3 (2.8) | -0.7 | -2.4 to 1.1 | 0.459 | 3.5 (2.7) | 3.5 (2.2) | 0 | -1.5 to 1.5 | 1.000 | 3.9 (2.8) | 3.4 (2.1) | -0.5 | -2 to 1.1 | 0.548 | 3.6 (2.6) | 2.3 (2.2) | -1.4 | -2.9 to 0.2 | 0.084 |
| EORTC Global Health status | 60.9 (24.4) | 54.3 (25.4) | -6.5 | -21.3 to 8.3 | 0.379 | 65.5 (24.5) | 60.2 (23.3) | -5.2 | -20 to 9.5 | 0.475 | 59.8 (22.2) | 59.2 (25.1) | -0.7 | -15.4 to 14.1 | 0.926 | 74.2 (16.6) | 70.2 (17.2) | -4 | -14.9 to 6.8 | 0.456 |
| EORTC Functional | 76.5 (18.5) | 76.4 (18.9) | -0.1 | -11.2 to 11 | 0.983 | 82.3 (13.7) | 82 (14.5) | -0.3 | -8.9 to 8.3 | 0.942 | 83.7 (11.5) | 81.6 (14.1) | -2.1 | -10.1 to 5.9 | 0.598 | 83.5 (12.9) | 86.3 (12.6) | 2.8 | -5.4 to 11 | 0.495 |
| EORTC Symptom | 21.9 (15.3) | 22.5 (17.1) | 0.6 | -9.1 to 10.2 | 0.905 | 18.4 (14.1) | 16.6 (14.1) | -1.8 | -10.4 to 6.7 | 0.666 | 18 (13.4) | 19.6 (13.9) | 1.7 | -6.8 to 10.2 | 0.692 | 13.3 (7.3) | 15.9 (12.7) | 2.6 | -4 to 9.1 | 0.430 |
| TUG | 11.5 (8.9) | 9.6 (3.6) | -1.9 | -6 to 2.3 | 0.363 | 8.3 (2.4) | 8.2 (2.5) | -0.1 | -1.7 to 1.4 | 0.853 | 9.1 (3.3) | 7.7 (1.7) | -1.3 | -3.1 to 0.4 | 0.124 | 8 (2.4) | 7.4 (2.1) | -0.6 | -2.1 to 0.9 | 0.416 |
| 6MWT | 501.9 (116.6) | 448.9 (111.5) | -53 | -138.3 to 32.3 | 0.214 | - | - | - | - | - | - | - | - | - | - | 508.3 (96) | 520.8 (90.6) | 12.5 | -53 to 77.9 | 0.700 |
